# What is the Future of the Gut Microbiota-Related Treatment? Toward Modulation of Microbiota in Preventive and Therapeutic Medicine

**DOI:** 10.3389/fmed.2014.00019

**Published:** 2014-07-10

**Authors:** Hubert Zatorski, Jakub Fichna

**Affiliations:** ^1^Department of Biochemistry, Medical University of Lodz, Lodz, Poland

**Keywords:** probiotic, IBD, gut microbiota, future treatment of gastrointestinal diseases, fecal transplant

## Introduction

The human body is colonized by a wide variety of micro-organisms, which are known to play an important role in regulation of metabolic functions and maintaining immune homeostasis. Instantaneously after birth, skin surface, oral cavity, and gut are settled by extensive range of microbes, mainly bacteria, but also archaea, fungi, viruses, and protozoa. Commonly called human microbiota, it consists of trillions of organisms from over 1000 species. The most abundant species are members of the phyla *Bacteroidetes* and *Firmicutes*. An important number of studies have been undertaken to determine if the gut microbiota is a promising target for clinical treatment or prevention. In the current issue of Frontiers in Medicine – Gastroenterology, Nagpal et al. paid close attention to the role of gut in health and disease. In their paper, they raise important questions and pay particular attention to ambiguities, which still remain to be elucidated to proper understanding of the complexity of human–microbe relationships. To better translate and apply current knowledge in clinical practice, answering the queries about compositional alterations in gut microbiome by diet or during diseases is necessary.

## Role of Gut Microbiota in Health and Disease

Bacterial diversity changes with age (1). As stated earlier, the adult gut ecosystem is dominated particularly by *Firmicutes* and *Bacteroidetes* phyla, but also by *Actinobacteria, Proteobacteria*, and *Verrucomicrobia* (2, 3); the anaerobic bacteria include *Bifidobacterium* spp and *Faecalibacterium* spp. It is important to emphasize that oxygen-tolerant bacteria, such as *Lactobacillus* spp. and members of *Proteobacteria* phylum may also appear in the gut, but in a low number. The population of aerobic bacteria is fluctuating over time and depends on diet and other environmental factors, such as hygiene, climate, geography, and ethnicity (4). The influence of nutrients on microbiome diversity was affirmed in the study by De Filippo et al. (5), in which Western diet in European children, high in animal protein and fat, caused reduction in short chain fatty acid-producing bacteria and increased the number of potentially pathogenic organisms in comparison to low animal protein diet and high-carbonate used by children in rural Africa. Nagpal et al. exposed the importance of diet in microbiota gentle alterations and the necessity of defining the ideal dietary macronutrients composition (ratios and types of carbohydrates, fats, and proteins).

Changes in gut microbiota in various types of diseases, such as obesity, IBD, type 2 diabetes, and non-alcoholic fatty liver disease is another problem raised by Nagpal et al. Gut microbiota dysbiosis causes increased gut permeability, systemic inflammation, and insulin resistance. Recent studies showed that patients suffering from IBD harbor altered gut microbiota, especially a reduced bacteria diversity (6, 7). In line, a decreased number of the butyrate-producing bacteria *Roseburia hominis* and *Faecalibacterium prausnitzii* have been observed in ulcerative colitis patients in comparison to control individuals (8). Of note, gut microbiota dysbiosis and lower levels of butyrate-producing bacteria were also observed in diabetes type 2 patients (9).

## Gut Microbiota as a New Treatment Target

Nagpal et al. underlined that numerous studies are conducted to indicate the therapeutic role of dietary supplements on gut microbiota and only after a precise identification of the role of each gut microbial family we could manipulate it by reducing the type and number of harmful and increasing beneficial populations. Several known therapeutics, such as prebiotics, probiotics, and antibiotics, can alternate the microbiota diversity.

Probiotics are defined by WHO as “live organisms, which – when administered in adequate amounts – confer a health benefit on the host.” The main micro-organisms used as probiotics are *Lactobacillus* and *Bifidobacterium*. Several studies suggest that probiotics improve intestinal barrier function, modulate the immune system, and enhance the host defense system against pathogens (10). In addition, probiotic intervention plays a positive role in prevention or improvement of some gastrointestinal diseases, such as IBD (11) and necrotizing enterocolitis (12).

Prebiotics are non-viable food components that confer health benefits on the host associated with modulation of microbiota (13). Thus prebiotics are dietary compounds, particularly insulin, fructooligosaccharides, and glucooligosaccharides, which have an impact on local gut bacterial microbiota. In the gut, prebiotics enhance the colonization of beneficial bacteria, such as *Lactobacillus* and *Bifidobacterium* and also stimulate immunity and prevent the adhesion of pathogens (10).

Finally, in their paper Nagpal et al. raise an important issue of the fecal transplant, which may become the therapy of the future. Fecal transplantation is a process of transferring fecal bacterial communities from healthy individuals to a recipient, whose microbiota have been disrupted. Nowadays, fecal transplantation has been used to treat recurrent *Clostridium difficile* infection by restoring the microbiota whipped out by the use of antibiotics. Even though a variety of problems in establishing the state of the art technique in fecal transplantation exist, the results are promising. Systematic literature review by Gough et al. to examine the effect of fecal transplant on 317 *Clostridium difficile* infected patients revealed that disease was resolved in 92% of cases (9).

## Conclusion

The gut microbiota is a complex ecosystem, which in a healthy state provides major functions to the host by influencing metabolism, modulating the immune system, and protecting against pathogens. Nowadays, the most important research task is to gain a deeper understanding of the complex relationships between the gut microbiota and disease. Nagpa et al. clearly show that gut microbiota is a promising target for preventive and therapeutic treatment and in a near future we will be able to use individual microbiome profiles in clinical practice to evaluate the current state and function of the gut. The use of prebiotics and probiotics, as well as in future the fecal transplant gives the clinicians a unique tool to treat chronic conditions, such as IBD, obesity and type 2 diabetes.

## Conflict of Interest Statement

The authors declare that the research was conducted in the absence of any commercial or financial relationships that could be construed as a potential conflict of interest.
